# Genetic variability in *CYP3A4 *and *CYP3A5 *in primary liver, gastric and colorectal cancer patients

**DOI:** 10.1186/1471-2407-7-118

**Published:** 2007-07-02

**Authors:** Guillermo Gervasini, Elena García-Martín, José M Ladero, Rosa Pizarro, Javier Sastre, Carmen Martínez, Monserrat García, Manuel Diaz-Rubio, José AG Agúndez

**Affiliations:** 1 Department of Pharmacology & Psychiatry, University of Extremadura, Badajoz, Spain; 2 Department of Biochemistry & Molecular Biology & Genetics, University of Extremadura, Badajoz, Spain; 3 Service of Gastroenterology, Hospital Clínico San Carlos, Complutense University, Madrid, Spain; 4 Service of Oncology Hospital Clínico San Carlos, Complutense University, Madrid, Spain

## Abstract

**Background:**

Drug-metabolizing enzymes play a role in chemical carcinogenesis through enzymatic activation of procarcinogens to biologically reactive metabolites. The role of gene polymorphisms of several cytochrome P450 enzymes in digestive cancer risk has been extensively investigated. However, the drug-metabolizing enzymes with the broader substrate specificity, CYP3A4 and CYP3A5, have not been analyzed so far. This study aims to examine associations between common CYP3A4 and CYP3A5 polymorphisms and digestive cancer risk.

**Methods:**

CYP3A4 and CYP3A5 genotypes were determined in 574 individuals including 178 patients with primary liver cancer, 82 patients with gastric cancer, 151 patients with colorectal cancer, and 163 healthy individuals.

**Results:**

The variant allele frequencies for patients with liver cancer, gastric cancer, colorectal cancer and healthy controls, respectively, were: CYP3A4*1B, 4.8 % (95% C.I. 2.6–7.0), 3.7 % (0.8–6.6) 4.3% (2.0–6.6) and 4.3% (2.1–6.5); CYP3A5*3, 91.8 % (93.0–97.4), 95.7% (92.6–98.8), 91.7% (88.6–94.8) and 90.8% (87.7–93.9). The association between CYP3A4*1B and CYP3A5*3 variant alleles did not significantly differ among patients and controls. No differences in genotypes, allele frequencies, or association between variant alleles were observed with regard to gender, age at diagnosis, tumour site or stage.

**Conclusion:**

Common polymorphisms on *CYP3A4 *and *CYP3A5 *genes do not modify the risk of developing digestive cancers in Western Europe.

## Background

The identification of low penetrance genes able to increase the risk of developing cancer could constitute a major tool for the identification of individuals with inheritable altered susceptibility. In this regard, the role of drug-metabolizing enzymes in cancer risk has been the object of several hundreds of studies performed over the last decade [1]. The main hypothesis underlying the link between drug-metabolizing enzymes and chemical carcinogenesis is based on the enzymatic activation of procarcinogens to biologically reactive metabolites. These reactive metabolites would interact with DNA, thereby causing altered gene expression or function, and eventually carcinogenesis. The primary metabolism of a variety of xenobiotic carcinogens is mainly mediated by cytochrome P450 (CYP) enzymes belonging to the CYP 1, 2 or 3 families, which together comprise 25 different isoenzymes. Among them, the most relevant are CYP1A1, CYP1A2, CYP1B1, CYP2E1, CYP3A4 and CYP3A5 [2-4]. Most of the latter enzymes are polymorphic, mutated alleles causing abolished, reduced or altered enzyme activity. To date, several studies have focused on the role of gene polymorphisms or enzyme activities of CYP1A1 [5-11], CYP1A2 [7,12-14], CYP1B1 [7,15] and CYP2E1 [6,16-19] in digestive cancer risk. Nevertheless, the impact on cancer risk of polymorphisms of the CYP enzymes with the broader substrate specificity, namely CYP3A4 and CYP3A5, has not been analyzed in detail so far.

CYP3A4 and CYP3A5 enzymes are the major enzymes for drug metabolism in adults [20], both enzymes making up nearly 30% of the total CYP enzymes expressed in the human liver [21]. Since no individualized quantification of CYP3A4 and CYP3A5 *in vivo *is presently possible due to a wide substrate specificity overlap, the joined enzyme activities are designated as CYP3A [22]. Both CYP3A4 and CYP3A5 are expressed in liver, stomach, colorectal epithelium and in colorectal cancer tissue [23-26], although large interindividual differences exist in the expression of both enzymes [27-29]. Besides a local effect of the enzyme activities in the metabolism of carcinogens, CYP3A4/5-dependent metabolism in the proximal intestine is likely to affect the luminal or vascular delivery of carcinogenic molecules to the liver or the colon.

These facts provide a plausible hypothesis for organ-specific dietary procarcinogen activation and therefore to digestive cancer risk. The hypothesis that justify the present study is based in the fact that CYP3A4 and CYP3A5 enzyme activities are involved in the activation of several procarcinogens, including aflatoxin B1 stergmatocystin, food-derived heterocyclic amines, alpha-hydroxytamoxifen and N'-nitrosonornicotine, and that both enzymes are expressed to a high level in the digestive tract [27,30-36]. Thus, it can be hypothesized that genetic differences causing variability in the regulation, expression or activity of CYP3A enzymes would be relevant factors modifying cancer susceptibility or clinical outcome. These allele-disease associations have already been shown for CYP3A gene polymorphisms and prostate, breast and lung cancer [37-43], albeit with controversial results. Although CYP3A4 and CYP3A5 play a relevant biological role in liver and in gut epithelium, no studies have addressed whether polymorphism of these enzymes are related to digestive cancer risk, probably because variant alleles for the corresponding genes have just recently been described. In this regard, novel findings show that *CYP3A5 *genotypes leading to high enzyme activity are related to oesophageal cancer [44]. However the interaction of *CYP3A4 *and *CYP3A5 *gene polymorphisms and the risk of developing the major digestive cancers such as liver, stomach or colorectal cancers remain unexplored. This study aims to analyze such interaction.

## Methods

The study group consisted of 411 unrelated patients with digestive cancers, including 178 patients with primary liver cancer, 82 patients with gastric cancer and 151 patients with colorectal cancer, and 163 healthy subjects. Table I shows a summary of the study groups included in the study. All the participants were white Spanish individuals, living in the same areas as the patients (Madrid and surrounding areas), and were included in the study after giving informed written consent. All the patients diagnosed with liver, stomach or colorectal cancer that attended the collaborating Services of the selected Hospitals were included in the study. The diagnosis was based on histology analyses of endoscopic biopsies and/or surgical resection specimens. Data regarding known previous digestive diseases, alcohol and tobacco consumption, serum tests for hepatitis B and C virus and other diseases were collected. Heavy drinkers were defined as individuals drinking more than 50 g of alcohol per day.

**Table 1:** Summary of the study groups.

	**NO**	**MALES**	**FEMALES**	**MEAN AGE (SD; RANGE)**
**Liver cancer**	178	145	33	66.1 (9.8; 20–88)
**Gastric cancer**	82	54	28	67.7 (13.9; 31–99)
**Colorectal cancer**	151	82	69	66.3 (11.2; 33–89)
**Healthy subjects**	163	111	52	46.3 (12.7; 21–95)

No: number of subjects

All the patients were requested to participate in the study, and all of them agreed to do so. Control samples were obtained from medical students, University and Hospital staff. A medical examination was made to identify subjects in good health. Over 95% of the healthy subjects requested agreed to participate in the study. The protocol was approved by the Ethics Committee of the San Carlos University Hospital, Madrid. A possible confounding factor in the present study is that, within a study group, the frequency of individuals carrying a determined variant allele may change with age in the event that the presence of such allele would be related to severe diseases. If a determined genotype has a "protecting effect" against any disease, it may be expected that in populations composed of older subjects there is an increased frequency of such a protecting genotype. Therefore we included within the control group a selected subgroup of 41 healthy subjects with ages ranging from 90 to 95 years [45]. The analysis of the *CYP3A4 *and *CYP3A5 *genotype indicates frequencies for genotypes that were identical to those of younger healthy subjects. The frequencies for CYP3A4*1B and CYP3A5*3 alleles were similar for younger and older controls (see Results section). 4.33% and 90.7% for younger controls and 4.18 and 91.2% for older controls. Another possible confounder is related to the fact that control subjects are highly educated people, and differences in lifestyle as compared to cancer patients may be expected. Since digestive cancers are partly related to diet, these changes in lifestyle may be relevant. However, it should be stated that patients and controls were interviewed to assure that diet and lifestyle do no differ between patients and control subjects.

Blood samples from all participants were stored at -80°C until analysis. The genomic DNA was prepared from peripheral leucocytes, and dissolved in sterile 10 mM Tris-HCl, pH 8.0, 1 mM ethylenediaminetetraacetic acid, at a final concentration of 400 to 600 mg per ml. DNA samples were purified according standard procedures [46] and stored at 4°C in sterile plastic vials.

The analyses for the CYP3A4*1B and CYP3A5*3 gene variants were carried out by amplification-restriction procedures as described elsewhere [47,48]. These allelic variants were analyzed instead of others because these are common variants that cause functional changes. [47,48].

### Statistical analysis

The study was designed taking into consideration the expected frequency of individuals expressing CYP3A5, i.e. carrying at least one CYP3A5*1 allele, which represents 13% of the Caucasian population [20]. The design permits the identification as statistically significant of a 1.7-fold increase or a 2-fold decrease in the frequency for CYP3A5 expressers among cancer patients. Statistical power was evaluated with a genetic model to analyze the frequency for carriers of the disease gene with an RR value = 2.5 (α = 0.05). The power calculated for associations with cancer risk for the presence of the CYP3A4*1B and CYP3A5*3 alleles is 90.2% and 99.3% for liver cancer, 74.5% and 93.9% for gastric cancer and 87.7% and 98.8% for colorectal cancer, respectively. The intergroup comparison values were calculated by using the statistical package SPSS 11.0.1. (SPSS Inc. Chicago, Ill, USA). The Chi-square (X^2^) test was used for comparison of genotype frequencies and association of *CYP3A4 *and *CYP3A5 *variant alleles, unless the conditions for the application of this test were not adequate. In such cases, Fisher's exact test was used to calculate the *p *value.

## Results

Seven different combinations of *CYP3A4 *and *CYP3A5 *genotypes were identified in the study groups. The summary of the genotypes is shown in Table 2. The comparison of genotype frequencies among digestive cancer patients and healthy controls indicates a similar genotype frequency across the study groups. No statistically significant differences were observed. The variant allele frequencies, as calculated from genotypes shown in Table 2, were almost identical for all study groups: CYP3A4*1B was present with allele frequencies of 4.8 (95% CI 2.6–6.9), 3.7 (95% CI 0.8–6.5) and 4.3% (95% CI 2.0–6.6) for liver, stomach and colorectal cancer patients, respectively, and 4.3% (2.1–6.5) for healthy individuals. The allele frequencies for CYP3A5*3 were 91.9 (95% CI 89.0–94.7), 95.7 (95% CI 92.6–98.8), and 91.7% (95% CI 88.6–94.8) for liver, stomach and colorectal cancer patients, respectively, and 90.8% (95% CI 87.7–93.9) among healthy individuals. No statistically significant differences in the allele frequencies were observed, although the frequency for the functional CYP3A5*1 allele among stomach cancer patients is lower (approximately 50%) than those observed for the rest of the study groups (p = 0.051 as compared to healthy subjects).

**Table 2:** *CYP3A4 *and *CYP3A5 *genotypes in digestive cancer patients and healthy control individuals.

***CYP3A4***	***CYP3A5***	**Liver cancer** **N (Frequency; 95% C.I.)**	**Stomach cancer** **N (Frequency; 95% C.I.)**	**Colorectal cancer** **N (Frequency; 95% C.I.)**	**Control subjects** **N (Frequency; 95% C.I.)**
*1A/*1A	*3/*3	144 (80.9; 75.1–86.7)	74 (90.2; 83.8–96.7)	121 (80.1; 73.8–86.5)	134 (82.2; 76.4–88.1)
*1A/*1A	*1/*3	17 (9.6; 5.2–13.9)	2 (2.4; 0–5.8)	18 (11.9; 6.8–17.1)	14 (8.6; 4.3–12.9)
*1A/*1A	*1/*1	0	0	0	1 (0.6; 0–1.8)
*1A/*1B	*3/*3	5 (2.8; 0.4–5.2)	1 (1.2; 0–3.6)	5 (3.3; 0.5–6.2)	1 (0.6; 0–1.8)
*1A/*1B	*1/*3	12 (6.7; 3.1–10.4)	5 (6.1; 0.9–11.3)	6 (4.0; 0.9–7.1)	12 (7.4; 3.3–11.4)
*1A/*1B	*1/*1	0	0	0	1 (0.6; 0–1.8)
*1B/*1B	*1/*3	0	0	1 (0.7; 0–2.0)	0

Both *CYP3A4 *and *CYP3A5 *genotypes were at Hardy-Weinberg's equilibrium among cases and controls and variant allele frequencies are consistent with those reported for both, European and American Caucasians [20,49]. Regarding the association between the CYP3A4*1B and CYP3A5*1 alleles, the proportion of individuals carrying both variants is much higher for all the study groups (6.7%, 6.1% and 4.6% for liver, gastric or colorectal cancer patients and 7.4% for control subjects) than expected from a random association of these alleles (0.3 to 0.8% of individuals, as calculated from actual allele frequencies in the study groups). These findings are in agreement with a previous study that reports such an SNP association in another Caucasian population with a frequency of 7.1%. [22,43]. Among patients, age at diagnosis was similar in all subgroups of patients, and this parameter was not related to the genotypes. Regarding control subjects, the frequencies for CYP3A4*1B and CYP3A5*3 alleles were 4.33% and 90.7% for younger controls and 4.18 and 91.2% for older controls. Gender, previous surgical therapy or chemotherapy, alcohol abuse or tobacco use did not influence the distribution of the polymorphisms studied among patients. Among liver cancer patients no differences in the distribution of polymorphisms regarding chronic infection with hepatitis B or C virus were observed. Among gastric cancer patients no statistically significant differences were observed regarding the anatomical site of the tumour. In contrast, significant differences showing an increase in the frequency of carriers of the CYP3A4*1A plus CYP3A5*3 haplotype, were observed among patients with intestinal-type gastric cancer as compared to healthy controls (95.3% versus 82.8%, p < 0.05).

Colorectal cancer patients were divided into three subgroups according to the anatomical site of the tumour (non-sigmoid colon, sigmoid colon and rectum), because differences in the association of other polymorphisms of drug-metabolizing enzymes related to tumour site have been reported [50,51]. The results are summarized in Table 3. An excess of patients with non-sigmoid colon cancer carrying the combined genotype CYP3A4*1A/*1B plus CYP3A5*3/*3 was observed as compared to healthy subjects (Chi-square = 10.3, p = 0.0015, non significant in multiple comparison analyses) With regard to allele frequencies, CYP3A4*1B allele frequency was 6.6%, 5.9% and 1% and CYP3A5*3 frequency was 91.1%, 90.1% and 93.6% among patients with non-sigmoid colon, sigmoid colon and rectum, respectively. The proportion of individuals carrying CYP3A4*1B and CYP3A5*1 alleles was 13.3%, 9.8% and 1.8% for patients with non-sigmoid colon, sigmoid colon and rectum, respectively. None of these differences were statistically significant. No differences regarding Dukes' stage of the tumour were observed.

**Table 3:** *CYP3A4 *and *CYP3A5 *genotypes in colorectal cancer patients according tumor site.

***CYP3A4***	***CYP3A5***	**NON-SIGMOID** **N (FREQUENCY; 95% C.I.)**	**SIGMOID** **N (FREQUENCY; 95% C.I.)**	**RECTUM** **N (FREQUENCY; 95% C.I.)**
*1A/*1A	*3/*3	33 (73.3; 60.4–86.3)	40 (78.4; 67.1–89.7)	48 (87.3; 78.5–96.1)
*1A/*1A	*1/*3	6 (13.3; 3.4–23.3)	6 (11.8; 2.9–20.6)	6 (10.9; 2.7–19.1)
*1A/*1B	*3/*3	4 (8.8; 0.6–17.2)*	1 (2.0; 0–5.8)	0
*1A/*1B	*1/*3	2 (4.4; 0–10.5)	3 (5.9; 0–12.3)	1 (1.8; 0–5.3)
*1B/*1B	*1/*3	0	1 (2.0; 0–5.8)	0

*p < 0.0015 compared to healthy subjects

## Discussion

Metabolism related to CYP enzymes differ between organs and tissues, and therefore it is expected that tissue specificity towards cancer-inducing substances is related to site-specific expression of xenobiotic-metabolizing enzymes. Digestive cancers are strongly related to dietary factors, and mechanisms involving the interaction of such dietary factors with determined polymorphisms of xenobiotic-metabolizing enzymes have been proposed [10]. Most of the *CYP *genes expressed in human gut and liver, namely *CYP1A1*, *CYP1A2*, *CYP1B1*, *CYP3A4 *and *CYP3A5 *[25], are of great relevance for carcinogen activation. With the exception of *CYP3A4 *and *CYP3A5*, all genes coding for these enzymes have been studied with regard to digestive cancer risk [1]. This study was aimed to fill the gap in the present knowledge of the putative role of polymorphisms of the two remaining relevant CYP enzymes in the digestive tract.

CYP3A enzyme activity shows interindividual variability due to the combined effect of genetics and interaction with drugs or environmental chemicals [22]. Both *CYP3A4 *and *CYP3A5 *genes are polymorphic and several variant alleles have been described for either the *CYP3A4 *or the *CYP3A5 *gene (for an updated list of alleles, see [52]). Only two variant alleles that are at linkage disequilibrium, namely CYP3A4*1B and CYP3A5*3 are common across diverse ethnic populations and have functional relevance. Other variant alleles are either extremely rare or irrelevant for enzyme activity [20], and therefore they were not analyzed in this study.

Present evidences indicate that CYP3A4*1B and CYP3A5*3 are functional polymorphisms *in vivo*. Regarding CYP3A4*1B, it seems to modify the ability to metabolize some CYP3A substrates, such as quinine [53], although it does not influence the metabolism of other substrates such as midazolam or dextromethorphan [49,54]. CYP3A5*3 is the commonest *CYP3A5 *allele and is associated with severely decreased enzyme activity [20].

The present study provides novel findings on the putative role of *CYP3A4 *and *CYP3A5 *polymorphisms in digestive cancer risk. In spite of the presence of CYP3A enzyme activities in gut and liver, and of the relevant role of such enzyme activities in carcinogen activation, our results do not support a major link between common *CYP3A4 *and *CYP3A5 *polymorphisms and digestive cancer risk. Nevertheless the possibility for a low to moderate effect for these polymorphisms especially in the smallest group, stomach cancer, can not be ruled out. Minor differences in the frequencies for individuals carrying the CYP3A4*1A plus CYP3A5*3 haplotypes were observed in some subgroups of gastric or colon cancer, as compared to healthy subjects, with p values under 0.05. These differences cannot be considered statistically significant in multiple-comparison analyses, and do not seem to have a relevant clinical impact. However, this is a topic that deserves investigation and further studies should focus on the role of such polymorphisms in liver, gastric or colorectal cancer in populations with extreme incidence rates of these cancers.

## Conclusion

Taking together the findings reported in the present study, it can be concluded that common polymorphisms of CYP3A enzymes with functional relevance are not significant factors in digestive carcinogenesis in Western Europe. This is in contrast to other human cancers that are related to such polymorphisms. Since human guts are the primary tissues that have contact with dietary carcinogens, it is intriguing that digestive cancer risk is not modified by genetic factors that are related to other human cancers such as prostate, breast or lung, thus reinforcing the hypothesis that the association of *CYP3A *polymorphisms with prostate or breast cancer are related to endogenous substances rather than xenobiotics carcinogens [37-43].

## Competing interests

The author(s) declare that they have no competing interests.

## Authors' contributions

GG, EGM, RP, CM and MG made substantial contributions in data acquisition, molecular genetic analyses, statistical analyses and data interpretation, and helped in manuscript preparation.

JML, JS and MDR were involved in the selection, evaluation and care of patients and helped in manuscript preparation.

JAGA conceived the study, reviewed the literature and drafted the manuscript.

All authors read and approved the manuscript.

## Pre-publication history

The pre-publication history for this paper can be accessed here:


